# Prognostic value of a patient-reported functional score versus physician-reported Karnofsky Performance Status Score in brain metastases

**DOI:** 10.3332/ecancer.2017.779

**Published:** 2017-11-10

**Authors:** Jai Prakash Agarwal, Santam Chakraborty, Sarbani Ghosh Laskar, Naveen Mummudi, Vijay M Patil, Kumar Prabhash, Vanita Noronha, Nilendu Purandare, Amit Joshi, Sandeep Tandon, Jitendra Arora, Rupali Badhe

**Affiliations:** 1 Department of Radiation Oncology, Tata Memorial Hospital, Parel, Mumbai 400012, India; 2 Department of Medical Oncology, Tata Memorial Hospital, Parel, Mumbai 400012, India; 3 Department of Radiology, Tata Memorial Hospital, Parel, Mumbai 400012, India; 4 Department of Pulmonary Medicine, Tata Memorial Hospital, Parel, Mumbai 400012, India

**Keywords:** patient-reported outcomes, quality of life, functional scores, Karnofsky performance status, performance status, brain metastases, non-small-cell lung cancer

## Abstract

**Introduction:**

Our aim was to investigate the added prognostic value of a patient-reported functional outcome score over Karnofsky Performance Status (KPS) in patients with non-small-cell lung cancers (NSCLC) with brain metastases.

**Materials and methods:**

The baseline data are from a prospective cohort study involving 140 consecutive patients presenting at our institute. A patient reported performance status (PRPS) was obtained by summing the physical- and role-functioning scale scores of the EORTC QLQ C30 questionnaire. Nested cox proportional hazards models predicting survival were developed including both KPS and PRPS (full model), KPS only (KPS Model), and PRPS only (PRPS model). The incremental value of the addition of KPS or PRPS was ascertained using the likelihood ratio test, model adequacy index and integrated discrimination Improvement (IDI).

**Results:**

PRPS was an independent and statistically significant prognostic factor and had only a moderate degree of agreement with KPS. All models showed nearly the same discrimination and calibration accuracy, but the likelihood ratio test comparing the full model to the KPS model was significant (L.R. Chi^2^ = 5.34, *p* = 0.02). Model adequacy index for the KPS model was 85% versus 95% for the PRPS model. IDI when comparing the KPS model to the full model was 0.0279, while it was 0.008 for the PRPS model versus the Full model.

**Conclusions:**

Use of patient-reported functional outcomes like PRPS can provide the same prognostic information as KPS in patients of NSCLC with brain metastases.

**Highlights:**

- Patient-reported functional status (PRPS) has a moderate degree of agreement with KPS.
- PRPS is an independent and significant predictor of survival in brain metastases.
- PRPS can replace KPS without loss of prognostic information.

## Introduction

Performance status (PS) has been defined by the National Cancer Institute as ‘a measure of how well a patient is able to perform ordinary tasks and carry out daily activities’ [38]. The baseline PS of patients is one of the most important factors influencing their prognosis [2]. In the context of patients with brain metastases, the baseline PS is the key prognostic factor determining survival [13, 32–34]. This is exemplified by the fact that the baseline PS is included in the diagnosis-specific graded prognostic assessment (DS-GPA) score across different primary sites [33]. The physician-reported performance status is usually reported in the form of a summary score, and the two most commonly used scales are the Karnofsky performance status (KPS) [18], and the Eastern Cooperative Oncology Group Performance Status (ECOG-PS) scales [23]. Of these, KPS has been used in successive trials in brain metastases patients and is also used for validated prognostic indices like the GPA [33].

PS is usually estimated by health care professionals during the course of a routine health care visit. However, estimation of PS using the KPS or ECOG scales suffers from interobserver variation due to the subjective nature [7, 28, 31, 36] of the assessment scale. Further, given the subjective nature of assessment, it is not surprising that there is only a moderate degree of agreement between the PS score as assessed by the patient themselves and the physician [3, 5, 6, 17, 22].

It is well known that physicians and other health care workers’ judgement about subjective measures, like patient’s pain, quality of life, anxiety, depression, etc., is different from that of the patients themselves [30]. Given the inherent subjectivity in PS assessment and the previous reports of disagreement between physician and patient assessed PS, it is reasonable to question if the use of patient-reported functional status would allow us to get better prognostic estimates as compared to KPS.

Functional status can be ascertained through the functional domains of Health-Related Quality of Life (HR-QOL) instruments like the European Organisation for Research and Treatment of Cancer (EORTC) Quality of Life Questionnaire C-30 (QLQ-C30) [20]. This questionnaire comprises of 30 items, of which 15 items contribute to five functional scales looking at physical, role, emotional, cognitive and social functioning.

Between June 2012 to April 2015 we enrolled 140 consecutive patients with non-small-cell lung cancer (NSCLC) with brain metastases planned for palliative whole brain radiotherapy in a prospective cohort study (CTRI Number: CTRI/2013/01/003299). As a part of this study, patients received usual care and underwent quality of life assessment using the EORTC QLQ C30. The objective of the current study is to ascertain if the patient reported functional status as ascertained using the EORTC QLQ C30 provides additional prognostic information over and above KPS.

## Materials and methods

One hundred and forty (140) consecutive patients with NSCLC with brain metastases were enrolled in this IRB approved prospective cohort study after written informed consent (Clinical Trial Registry of India number: CTRI/2013/01/003299). The study was conducted between June 2012 to April 2015 at a tertiary cancer centre in India. The quality of life (QOL) assessments and mini-mental state examination (MMSE) [11] was performed at baseline and information regarding traditional prognostic variables was recorded. QOL was assessed using the EORTC QLQ C-30 [1], LC-13 [4], and BN-20 questionnaires [21]. All questionnaires were self-reported. All patients underwent palliative whole brain radiotherapy (WBRT) to a dose of 20 Gy in five fractions over one week. Similar QOL and MMSE assessments were also performed at subsequent follow-up. Data regarding baseline quality of life scores were available for all patients.

For the purpose of this study, we calculated the baseline values of the functional scales of the EORTC QLQ C30 questionnaire. The calculation was done as per the methodology suggested by the EORTC QLQ scoring manual [10]. This involved calculation of the raw score for each scale by obtaining the average score for all the items in the scale. This was followed by linear transformation of the raw score to obtain a score ranging between 0 and 100. For each functional scale, increasing scores represented a better functioning. As KPS primarily focusses on the physical functioning, we chose the physical- and role-functioning scales for constructing a Patient-Reported Performance Scale (PRPS). The seven items which are a part of this PRPS are shown in Table 1.

The scale to which each item belongs is also indicated. Items taken from the EORTC QLQ C30 questionnaire.

The PRPS was the average of the physical and role functioning scale scores. No further score transformation was done.

PRPS = (Physical Functioning Scale Score + Role Functioning Scale Score)/2 .... Equation 1.

The above calculation methodology was adopted, as it retained the original scale definition as well as the original score calculation method as defined by the EORTC. However, we also used another method of calculation where the PRPS was derived from the seven questions directly using the formulae:

*PRPS Raw = (Q1+Q2+ .... +Q7)/7 .......*(2)

*PRPS = [1 - ((PRPS Raw-1)/3)]x100 ....* (3)

However, the PRPS calculated using this alternate methodology did not alter the essential results in the study (results not shown).

Prior to analysing the utility of PRPS in predicting prognosis, we ascertained the degree of agreement between the PRPS and the KPS. Traditional measures to define agreement like the weighted kappa score are not useful in this setting as the two scales are different. Hence, we used polychoric correlation as a measure of agreement. Polychoric correlation allows estimation of agreement between two ordinal scales measuring an underlying latent construct which is assumed to be continuous [12]. After quantifying the degree of agreement between the KPS and PRPS, we ascertained the impact of the addition of PRPS as a prognostic factor. In order to ascertain the successive nested, cox proportional hazards models were fit. These models were as follows:

1. Full model: age, gender (male/female), number of brain metastases (1–3 or 3 or more), extracranial disease (present/absent), epidermal growth factor receptor (EGFR) mutation (mutated/wild type/not tested), KPS and PRPS.
2. KPS Model: Same as model 1 except PRPS was excluded.
3. PRPS Model: Same as model 1 except KPS was excluded.

Proportional hazards and linearity assumptions were checked for all models. In order to check for nonlinearity, continuous variables in the model (age, PRPS and KPS) were expanded using restricted cubic splines with four knots and ANOVA test was used to determine if linearity assumptions needed to be relaxed. The likelihood ratio test was used to determine whether the addition of PRPS or KPS resulted in a better model fit, with a *p* value of < 0.05 considered as statistically significant. Thus, the likelihood ratio test when comparing KPS model to full model, estimated if the inclusion of PRPS resulted in significantly better fit as compared to the KPS model. Similarly, the likelihood ratio test when comparing PRPS model to the full model, estimated the benefit of the addition of KPS to the PRPS model. A model adequacy index was also calculated, which is the ratio of the likelihood ratio of the subset model to that of the full model [16]. An adequacy index of 1 indicates that the prognostic information in the subset model is same as that of the full model, or in other words inclusion of the additional covariate is not needed.

Model discrimination was checked using the Harrell’s concordance index [16], time-dependent receiver operating curves (ROC) and the area under curve (AUC) calculated at various time points. The ROC estimates were derived using cumulative case/dynamic control method based on inverse probability-of-censoring weights as proposed by Uno et al [37]). Risk assessment plots were generated and Integrated Discrimination Index (IDI) were calculated using the method proposed by Pickering et al [26] using mortality prediction estimates at six months. Ninety-five per cent confidence intervals of the IDI were also calculated using 1000 bootstraps. Model calibration at six months (182.5 days) was checked using calibration plots. Model discrimination and calibration statistics were internally validated using bootstrapping (1000 bootstrap samples). Full details of the analytic methodology and accompanying analysis with comments are available in Appendix.

All analyses were conducted using R (version 3.3.3, Vienna, Austria) [27] in RStudio IDE (version 1.0.136, RStudio, Inc., Boston, MA, USA). Packages used for the analysis were polycor, rms, pec, rap, survAUC and survminer (cited in Appendix).

## Results

### Patient characteristics

**Figure 1.**
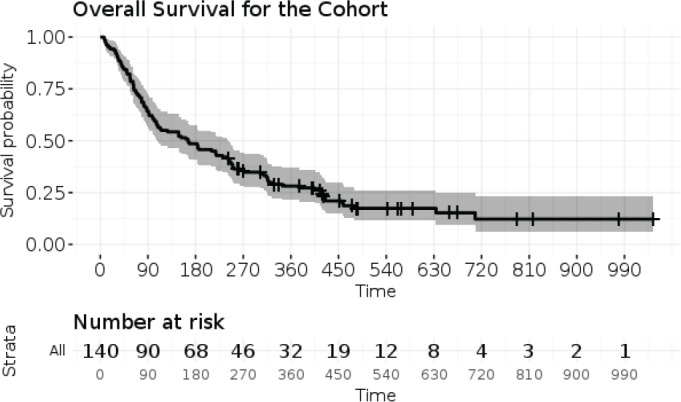
Kaplan–Meier survival curve for the entire population with 95% confidence intervals of the estimate. The number at risk are represented at each time interval are represented below the curve.

The demographic and disease-related characteristics of the patient population are described in Table 2. The database was closed for analysis in August 2016, by which time, 111 patients (79.3%) had died. The median overall survival calculated from the date of completion of WBRT was 166 days (95% CI: 108–242 days). All deaths were related to disease progression. The 30 day, 60 day and 120 day survivals were 92%, 79% and 55% (Figure 1). Assuming a rule of thumb of 10 events per variables, 111 events gave us sufficient power to examine 11 variables in a prognostic model [24, 25].

Figures in parentheses represent the percentage of patients in the given category for categorical variables and interquartile range (IQR) for continuous variables.

EGFR: epidermal growth factor receptor; IQR: interquartile range; KPS: Karnofsky Performance Status; MMSE: Mini-mental Status Examination Score Category; RPA: recursive partitioning analysis,

### Agreement between PRPS and KPS

**Figure 2.**
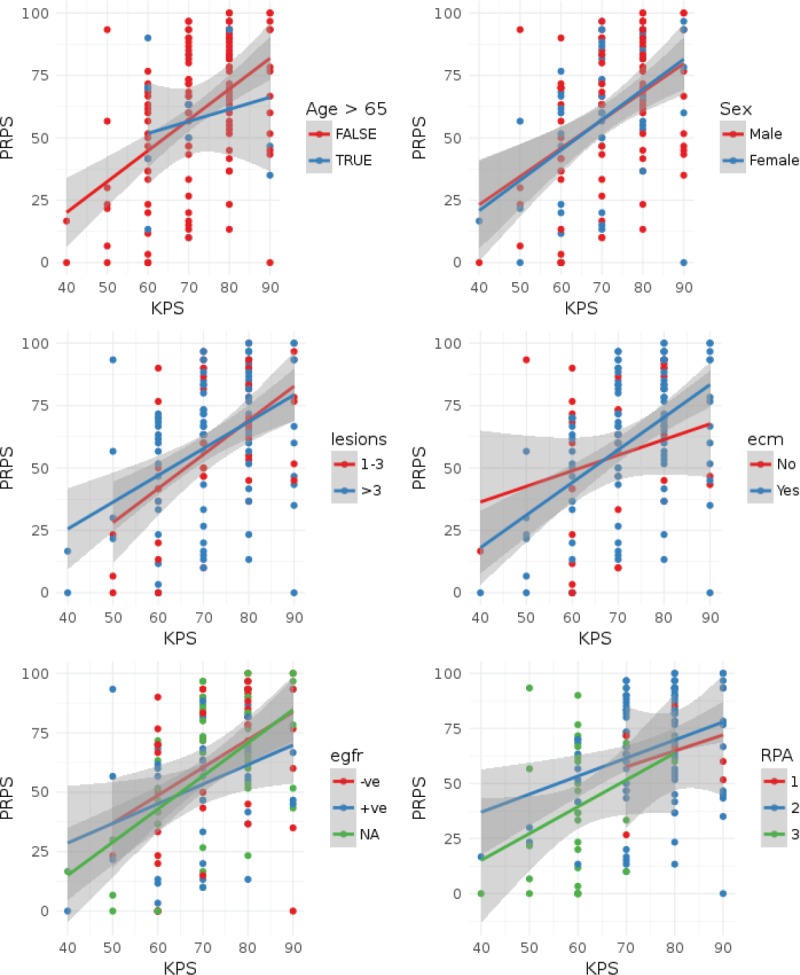
Scatter plots of PRPS score versus KPS for the patients in the study. Points have been color coded as per the important prognostic variables (age, sex, number of lesions, the presence of extracranial disease, EGFR status) and the RPA category. Lines represent linear regression fits and shaded bands the 95% confidence intervals of the same. In order to demonstrate the effect of age, we have categorised the age into two categories, > 65 years and ≤ 65 years. Lesions = Number of brain lesions (1-3 vs. > 3), ECM: extracranial disease, EGFR: epidermal growth factor receptor; RPA: recursive partitioning analysis class. Individual subset correlation coefficients can be seen in the Appendix.

The polychoric correlation coefficient between KPS and PRPS was 0.46 (standard error: 0.07) indicating that only a modest degree of agreement existed between KPS and PRPS. The test for bivariate normality was not significant (Chi-square = 159.8, df = 229, *p* = 0.9998) indicating that the assumptions of polychoric correlation were not violated. As can be seen in Figure 2, there is a wide variability between the PRPS and KPS. The important prognostic variables which have been included in the model did not seem to influence the correlation between PRPS and KPS (Figure 2 and Appendix). Patients with a poorer cognitive function in general had a better agreement between the KPS and PRPS scores, though the degree of agreement was still modest with the highest polychoric coefficient of 0.67 for patients with poor cognitive function as defined by the MMSE test (Appendix).

### Comparative evaluation of prognostic models

The three pre-specified models are presented in Table 3 and as can be seen, in the full model as well as the PRPS model, PRPS was found to be a significant predictor of survival. While KPS was a significant predictor of survival in the KPS model, it was not significant in the full model when PRPS was also included. Model assumptions were checked as shown in Appendix.

Contrasts have been depicted for continuous variables for a better understanding of the hazard ratios. Statistically significant variables in the model are indicated with^*^. p values < 0.05 is taken as significant and all coefficients have been rounded to two decimal places.

EGFR: epidermal growth factor receptor; KPS: Karnofsky performance status; LR Chi^2^: likelihood ratio chi-square value; PRPS: patient-reported performance scale. See Appendix for the graphical representation and the summary statistics for the individual models.

The likelihood ratio (LR Chi^2^) test comparing the full model and the KPS model was significant indicating that addition of PRPS improved the goodness to fit (LR Chi^2^ = 5.34, *p* = 0.02*). However, the likelihood test comparing the full model and the PRPS model was not significant, indicating that addition of KPS did not improve the goodness to fit significantly (LR Chi^2^ = 1.77, *p* = 0.18) when PRPS was already present in the model. The model adequacy index for the PRPS model was 95.2%, while for the KPS model it was 85.6%. In other words, the PRPS model explained about 95% of the predictive information contained in the full model, while the KPS model could explain only 85% of the same.

**Figure 3.**
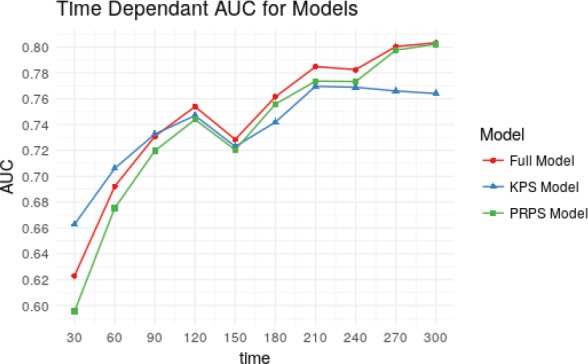
Line plots showing the time-dependent cumulative case/dynamic control ROC values for the three models plotted from times 30 days to 300 days.

The Harrell’s C-Statistic were 0.69, 0.68, and 0.68 for the full model, KPS model, and PRPS model respectively. Optimism corrected values (using 1000 Bootstrap samples) of the C-statistic were 0.66, 0.65, and 0.65, respectively. The time-dependent cumulative case/dynamic control-integrated AUC values for the three models were 0.72, 0.72, and 0.71 for the full, KPS and PRPS models, respectively. Figure 3 shows that actual time-dependent AUC values for the three models at different time points. As can be seen, the KPS model has slightly better discrimination ability at shorter follow-up times, while the converse is true about the PRPS model. However, on the whole, the discriminative ability of the PRPS model is nearly similar to that of the full model at all time points.

**Figure 4.**
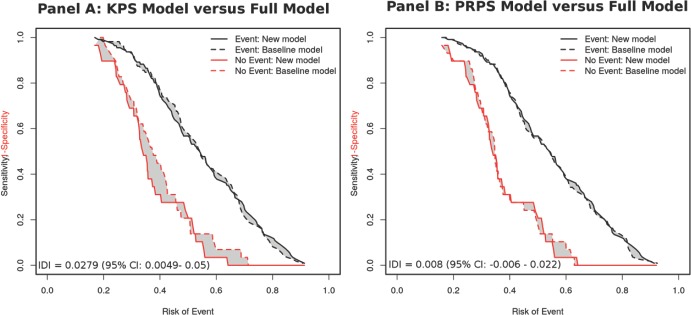
Risk assessment plots of the KPS model versus the full model (Panel A) and the PRPS model versus the full model (Panel B). IDI: integrated discrimination improvement. 95% CI: 95% confidence intervals of the estimate. A higher IDI indicates a better model predictive performance. Plots drawn from mortality estimates at six months.

The findings were confirmed in the risk assessment plots (RAP) and the values of integrated discrimination improvement (IDI) (Figure 4 and Appendix). While the addition of PRPS to a model with KPS resulted in a slight improvement in the discrimination between non-events, there was no difference in discrimination for patients with events. On the other hand, addition to KPS to a model with PRPS does not result in any discernible improvement in the predictive ability for either patients with or without events.

Bootstrap optimism–corrected calibration plots of the three models showed a good fit with mean absolute errors and 0.9 quantile of the absolute error being 0.035 (0.07), 0.036 (0.06), and 0.037 (0.08) for the full model, KPS model, and PRPS model, respectively. Model calibration plots are shown in the Appendix.

## Discussion

As a construct, performance status (PS) has both a subjective and an objective domain, but the current methods of assessment in the clinic use mainly subjective measures [19]. It is, therefore, surprising that physicians continue to rate the patient’s performance status, while other subjective issues, like pain and quality of life, are usually patient reported. The EORTC QLQ C30 has seven questions that deal with the physical and role function, and these questions are more specific as compared to traditional PS assessment methods like KPS or ECOG scales. Hence, intraobserver variability is likely to be less as compared to traditional PS assessment as the subjectivity arising out of physician assessment would be eliminated [35].

Guzelant et al have previously shown that of all the scales in the EORTC QLQ C30 questionnaire, physical functioning and role functioning scales have the strongest correlation with KPS [15]. In the current study, we demonstrate that a composite score of the physical and role functioning score (PRPS) provides valid prognostic information over and above KPS and may be used instead of KPS with little loss in model predictiveness and accuracy.

Previous studies evaluating physician- and patient-reported performance scales have shown significant disagreement between the two [3, 5, 6, 17, 22]. As shown in this study, there is only a modest agreement between patient-reported functional scores and physician-reported PS. In addition to inter-observer variability in rating PS [3], it is known assessment of PS is influenced by physician bias. For example, Broderick et al have demonstrated that younger patients are generally assigned more favourable PS scores by physicians [7]. This is despite the fact that PS does not correlate well with comorbidity and the disease stage [9].

While the current study demonstrates that PRPS can substitute for KPS without loss of prognostic information, results from previous studies have been conflicting. For example, Ando et al have reported that oncologist rated PS scores best fit the observed survival [3]. However, unlike the current study, the prognostic model comparisons were not done based on nested models and robust internal validation or calibration was not performed.

One of the concerns regarding the use of patient performance status has been that patients tend to rate their own performance status poorly [3, 8, 22, 29]. The reason behind this are poorly understood but may be related to depression [17] or a subconscious desire to seek help [3]. The use of patient-reported PS can result in almost half the patients excluding themselves from clinical trials where PS is a part of the inclusion criteria [8]. The use of PRPS as used in the present study can be a way around, wherein the patient-reported scores are used for determining prognosis, while physician-determined PS is used to determine clinical trial eligibility. Further use of specific questions as used in the EORTC QLQ C30 may reduce the possibility of patients rating their performance status artificially lower [35].

As Suh et al. have reported composite PS derived from a patient reported review of systems, can be obtained in a longitudinal fashion in the clinic and longitudinal changes in this influence prognosis [35]. Further, unlike the findings reported by Suh et al, we found that baseline PRPS was a significant and independent predictor of survival [35].

Our current study has several strengths in this regard. It is prospectively conducted in a cohort of patients with a single disease site. Consecutive patients were recruited to minimise selection bias. As all patients received the same radiotherapy treatment variability was also minimised which may have been a factor in previous studies [3, 5]. Follow-up was also complete and adequate. The final event (death) was observed in more than 80% of the patients. Baseline patient-reported QOL data were complete as was information regarding other prognostic variables. Further, all these information was collected in a standardised proforma, which further minimised interobserver variability. Hence, this data set was ideal to compare and contrast prognostic models that employ baseline predictive factors. The findings from the study lend further credence to the observation that patient-reported outcome measures are better predictors of survival as compared to traditional PS [14].

The limitations of the study include the fact that it was conducted in a single centre and most patients had an adenocarcinoma histology. Hence, applicability of this model to patients with other histologies and brain metastases from other sites may be limited. Nonetheless, performance status is a part of all diagnosis-specific GPA indices [33], which is an indicator of its importance in patients with brain metastases irrespective of the site of origin.

Further all patients in this study had received whole brain radiotherapy. Patients who received stereotactic radiotherapy for brain metastases, usually have a lower intracranial disease burden and hence likely to have better performance status. However, even in this setting, a score like the PRPS is likely to discriminate better between subtle grades of functional impairments as compared to the ECOG and KPS scales. As shown in the appendix, patients with better MMSE scores had a poorer correlation between the KPS and PRPS scale.

Patients’ assessment of their own HRQoL assessments is influenced by several factors like comorbidities, cognitive function, and response bias. However, the same issues affect assessment of a subjective domain like functional status when done by other observers. Eliminating other observers from the assessment process, has to potential to reduce inter-observer bias as the patient directly reports his/her functional status. In this regard, the finding that patients with poorer cognitive function had better agreement between the PRPS and the KPS score provides some insights. It is likely that the functional deficits produced in the presence of major cognitive deficits would have caught the attention of the treating physician resulting in better agreement in grading the functional deficits. In patients with better cognitive function (as assessed by the MMSE), the functional deficits would have been subtle and hence would have either not been noted by the physician or graded adequately using KPS.

## Conclusion

Our study shows that patient functional score derived from the EORTC QLQ C30 (PRPS) was both a statistically significant and an independent predictor of mortality in patients with brain metastases. It also gave the same prognostic information as KPS in our prognostic model. There was significant disagreement between PRPS and KPS, although the same were measured at the same time point. The results of this study highlight the importance of patient reported outcome measures in patients with brain metastases and should spur further research in the use of patient-reported functional outcomes in this population.

## Funding

The project was supported by an intramural grant from Tata Memorial Hospital.

## Conflicts of interest

None of the authors have any conflicts of interest to declare.

## Figures and Tables

**Table 1.** Items taken from the EORTC QLQ C30 used for calculating the PRPS.

Q No.	Item	Scale
1	Do you have any trouble doing strenuous activities, like carrying a heavy shopping bag or a suitcase?	Physical
2	Do you have any trouble taking a long walk?	Physical
3	Do you have any trouble taking a short walk outside of the house?	Physical
4	Do you need to stay in bed or a chair during the day?	Physical
5	Do you need help with eating, dressing, washing yourself or using the toilet?	Physical
6	Were you limited in doing either your work or other daily activities?	Role
7	Were you limited in pursuing your hobbies or other leisure time activities?	Role

**Table 2.** Basic demographic- and disease-related characteristics of the entire population.

Variables	Category	Number ( Total = 140)
Age	Median (IQR)	54 (45.8–60) years
Gender	Male	85 (60.7%)
	Female	55 (39.3%)
KPS	< 70	39 (27.9%)
	70–80	86 (61.4%)
	90	15 (10.7%)
Comorbidities	Yes	28 (20.0%)
	No	112 (80.0%)
Pathology	Adenocarcinoma	135 (96.4%)
	Non-adenocarcinoma	5 (3.6%)
Symptomatic brain metastases	Yes	127 (90.7%)
	No	13 (9.3%)
Motor deficits	Yes	26 (18.6%)
	No	114 (81.4%)
Cranial nerve palsy	Yes	13 (9.3%)
	No	127 (90.7%)
Number of brain metastases	1	6 (4.3%)
	2	25 (17.9%)
	3	12 (8.6%)
	4 or more	97 (69.3%)
RPA class	I	8 (5.7%)
	II	96 (68.6%)
	III	36 (25.7%)
EGFR mutation	Present	40 (28.6%)
	Absent	50 (35.7%)
	Not tested	50 (35.7%)
MMSE score category	No cognitive impairment (24–30)	72 (51.4%)
	Mild impairment (18–23)	41 (29.3%)
	Severe impairment (0–17)	27 (19.3%)

**Table 3.** Showing the model coefficients expressed as hazard ratios with 95% confidence intervals of the same shown inside parentheses.

Variable	Contrast	Full model	KPS model	PRPS model
Age	40:70	1.28 (0.71–2.32)	1.21 (0.67–2.16)	1.34 (0.75–2.39)
Gender	Male:Female	1.78 (1.17–2.71)*	1.87 (1.23–2.84)*	1.72 (1.14–2.62)*
Extracranial metastases	No:Yes	1.50 (0.97–2.34 )	1.51 (0.97–2.35)	1.53 (0.99–2.34)
Number of lesions	> 3:1–3	1.46 (0.95–2.22)	1.47 (0.96–2.33)	1.46 (0.96–2.23)
EGFR mutation	+ve:-ve	0.67 (0.41–1.12)	0.77 (0.47–1.26)	0.64 (0.39–1.06)
EGFR mutation	NA:-ve	1.50 (0.95–2.36)	1.60 (1.01–2.51)^*^	1.45 (0.93–2.30)
KPS	90:70	1.26 (0.90–1.79)	1.56 (1.15–2.11)^*^	NA
PRPS Score	80:40	1.41 (1.05–1.88)^*^	NA	1.55 (1.21–2.00)^*^
LR Chi^2^		37.15	31.81	35.37

## Appendix I

### PRPS calculation

As indicated in the manuscript the PRPS was calculated as the sum physical and role function scores obtained from the baseline EORTC QLQ C30 questionnaires.

**Table:** Physical Function Scale

Min.	1st Qu.	Median	Mean	3rd Qu.	Max.
0	38.33	60	57.05	80	100

**Table:** Role Function Scale Scores

Min.	1st Qu.	Median	Mean	3rd Qu.	Max.
0	33.33	66.67	61.31	100	100

**Table:** Patient reported Performance Scale

Min.	1st Qu.	Median	Mean	3rd Qu.	Max.
0	41.67	63.33	59.18	83.33	100

#### Agreement between PRPS and KPS

A plot of KPS against the PRPS shows significant degree of variability. The variability is demonstrated for other prognostic indicators like age, gender, RPA category, etc.

We will also calculate the Pearson correlation coefficient between KPS and PRPS to complement the graphical information above for different subgroups and for the whole dataset.

##
##  Pearson’s product–moment correlation##
## data:  bm$PRPS and bm$KPS
## t = 5.9193, df = 138, p value = 2.435e-08
## alternative hypothesis: true correlation is not equal to 0
## 95 per cent confidence interval:
##  0.3069995 0.5731030
## sample estimates:
##      cor
## 0.449984

**Table:**

FALSE	TRUE	
0.4832	0.1742	
Male	Female	
0.4366	0.4679	
1–3	> 3	
0.5445	0.4117	
No	Yes	
0.2433	0.504	
-ve	+ve	NA
0.4116	0.3498	0.5592
1	2	3
0.3271	0.3014	0.312
Mild	No	Severe
0.06933	0.5376	0.6451

As can be seen from the above, the correlation coefficient seems to vary between 0.17 and 0.56 with the largest correlations being observed for the EGFR unknown subgroup and the subset with 1–3 brain metastases. However, overall the correlation coefficients indicate only a weak–moderate correlation.

In order to formally acertain the magnitude of agreement between KPS and PRPS, we will use the polychoric correlation. The results of the polychoric correlation analysis is indicated below.

##
## Polychoric Correlation, 2-step est. = 0.4604 (0.06597)
## Test of bivariate normality: Chisquare = 159.8, df = 229, p = 0.9998

**Table:** Polychoric correlation coefficient for age groups

	FALSE	TRUE
**type**	polychoric	polychoric
**rho**	0.4902518	0.161044
**var**	0.004613493	0.05988673
**n**	121	19
**chisq**	139.7936	45.0513
**df**	224	44
**ML**	FALSE	FALSE

**Table:** Polychoric correlation coefficient for gender

	Male	Female
**type**	polychoric	polychoric
**rho**	0.4564041	0.4849123
**var**	0.007279636	0.01055285
**n**	85	55
**chisq**	133.7854	97.89713
**df**	189	159
**ML**	FALSE	FALSE

**Table:** Polychoric correlation coefficient for number of lesions

	1–3	> 3
**type**	polychoric	polychoric
**rho**	0.5270248	0.445308
**var**	0.01156839	0.006553012
**n**	43	97
**chisq**	82.61368	135.9663
**df**	111	204
**ML**	FALSE	FALSE
No	Yes	
0.1745	0.5164	

**Table:** Polychoric correlation coefficient for EGFR receptor status

	-ve	+ve	NA
**type**	polychoric	polychoric	polychoric
**rho**	0.4578586	0.3712709	0.5545893
**var**	0.01312181	0.01860321	0.008961416
**n**	50	40	50
**chisq**	89.13907	74.80448	97.55861
**df**	103	119	154
**ML**	FALSE	FALSE	FALSE

**Table:** Polychoric correlation coefficient for RPA class

	1	2	3
**type**	polychoric	polychoric	polychoric
**rho**	0.2853132	0.2990349	0.3232679
**var**	0.1476513	0.009558001	0.02769103
**n**	8	96	36
**chisq**	16.84401	126.7852	48.66904
**df**	13	199	87
**ML**	FALSE	FALSE	FALSE

**Table:** Polychoric correlation coefficient for cognitive impairment defined by MMSE score

	Mild	No	Severe
**type**	polychoric	polychoric	polychoric
**rho**	−0.02262085	0.5264594	0.6680286
**var**	0.02782513	0.007320261	0.01047318
**n**	41	72	27
**chisq**	89.31905	84.63891	58.79614
**df**	107	119	109
**ML**	FALSE	FALSE	FALSE

### Prognostic model discrimination and calibration

#### Model specification

First we will build the first model including both PRPS and KPS. The other explanatory variables to be included are:

1. Age (modelled as a continuous variable)
2. Gender (dichotomous variable)
3. KPS (modelled as as continous variable)
4. Extracranial Disease (dichotomous variable)
5. Number of mets (modelled as a factor variable: 1-3 mets and more than 3 mets)
6. EGFR mutation status (modelled as a factor variable:present, absent and unknown)
7. PRPS (modelled as continuous variable)

The three continuous variables would be expanded by restricted cubic splines using four knots. The choice of four knots for restricted cubic splines stems from the observation by Harrell et al, that using four knots offers an adequate fit of the model and is a good compromise between flexibility and loss of precision caused by overfitting a small sample.

#### Checking linearity assumption

##                Wald Statistics     Response: S
##
## Factor          Chi-Square d.f. P
## Age               4.81       3   0.1860
##  Non-linear       4.52       2   0.1042
## Sex               9.51       1   0.0020
## KPS               5.24       3   0.1549
##  Non-linear       2.84       2   0.2423
## PRPS              8.15       3   0.0431
##  Non-linear       2.14       2   0.3430
## ECM               1.24       1   0.2656
## lesions           3.21       1   0.0731
## EGFR             11.76       2   0.0028
## TOTAL NON-LINEAR 10.22       6   0.1156
## TOTAL            45.74      14   <.0001

As shown by the results of the AONVA the linearity assumptions seems to hold true.

Further confirmation is obtained by visual examination of the plots of martingale residuals against the continuous variables, which allows us the check the functional forms of the covariates for the linearity assumptions. As the loess smooth lines and the 95% confidence intervals show the lines are approximately centred around 0 with no discernable pattern indicating that we can proceed with a linear assumption.

#### Checking proportional hazards assumption

We now check the proportional hazards assumption using scaled shoenfeld residual both using hypothesis testing and graphical methods.

As can be seen from the plots above, the proportional hazards assumption holds true. The global test of proportional hazards penalised for the 8 degrees of freedom is non-significant with *p* value of 0.13 indicating that the proportional hazards assumption is not violated.

#### Outlier detection

In the plot below, we fit the deviance residuals to the observation ID to detect overtly influential outliers. As can be seen, there are no significant outliers detected. The loess smooth line is approximately centred around 0 with no definite pattern. Most observations lie within the 1 standard deviation.

From the above, we can see that the assumptions of the cox proportional hazards model hold true. The final full model can thus be specified as a linear combination of the covariates.

##### Full model summary

## Cox Proportional Hazards Model
##
##  cph(formula = S ~ Age + Sex + ECM + lesions + EGFR + KPS + PRPS,
##      data = dat, x = TRUE, y = TRUE, surv = TRUE, time.inc = 182.5)
##
##                       Model Tests       Discrimination
##                                            Indexes
##  Obs        140    LR chi2     37.15    R2       0.233
##  Events     111    d.f.            8    Dxy      0.371
##  Center -1.1611    Pr(> chi2) 0.0000    g        0.680
##                    Score chi2  39.33    gr       1.974
##                    Pr(> chi2) 0.0000
##
##              Coef     S.E.    Wald Z Pr(>|Z|)
##  Age         0.0083  0.0101    0.82  0.4104
##  Sex=Female −0.5770  0.2147  −2.69  0.0072
##  ECM=Yes    −0.4074  0.2250  −1.81  0.0703
##  lesions=>3  0.3757  0.2159    1.74  0.0818
##  EGFR=+ve   −0.3970  0.2583  −1.54  0.1243
##  EGFR=NA     0.4026  0.2328    1.73  0.0838
##  KPS        −0.0118  0.0088  −1.34  0.1814
##  PRPS       −0.0086  0.0037  −2.33  0.0197
##
##             Effects                  Response : S
##
##  Factor           Low    High  Diff. Effect   S.E.     Lower 0.95  Upper 0.95
##  Age              40     70     30    0.24841 0.30175  -0.343010   0.83982
##   Hazard ratio    40     70     30    1.28200      NA   0.709630   2.31600
##  KPS              90     70    -20    0.23554 0.17624  -0.109890   0.58097
##   Hazard ratio    90     70    -20    1.26560      NA   0.895940   1.78780
##  PRPS             80     40    -40    0.34445 0.14777   0.054837   0.63406
##   Hazard ratio    80     40    -40    1.41120      NA   1.056400   1.88530
##  Sex - Male:Female 2      1     NA    0.57697 0.21466   0.156250   0.99768
##   Hazard ratio     2      1     NA    1.78060      NA   1.169100   2.71200
##  ECM - No:Yes      2      1     NA    0.40736 0.22504  -0.033702   0.84843
##   Hazard ratio     2      1     NA    1.50280      NA   0.966860   2.33600
##  lesions - >3:1-3  1      2     NA    0.37569 0.21589  -0.047439   0.79881
##   Hazard ratio     1      2     NA    1.45600      NA   0.953670   2.22290
##  EGFR - +ve:-ve    1      2     NA   -0.39703 0.25829  -0.903280   0.10922
##   Hazard ratio     1      2     NA    0.67231      NA   0.405240   1.11540
##  EGFR - NA:-ve     1      3     NA    0.40259 0.23281  -0.053708   0.85888
##   Hazard ratio     1      3     NA    1.49570      NA   0.947710   2.36050

##### KPS model summary

The KPS model (model m2) is specified below. In this model PRPS was not included.

## Cox Proportional Hazards Model
##
##  cph(formula = S ~ Age + Sex + ECM + lesions + EGFR + KPS, data = dat,
##      x = TRUE, y = TRUE, surv = TRUE, time.inc = 182.5)
##
##                       Model Tests       Discrimination
##                                            Indexes
##  Obs        140    LR chi2     31.81    R2       0.203
##  Events     111    d.f.            7    Dxy      0.349
##  Center -1.4674    Pr(> chi2) 0.0000    g        0.640
##                    Score chi2  33.06    gr       1.897
##                    Pr(> chi2) 0.0000
##
##             Coef    S.E.   Wald Z Pr(>|Z|)
##  Age         0.0062 0.0100   0.63 0.5317
##  Sex=Female −0.6259 0.2141 −2.92 0.0035
##  ECM=Yes    −0.4109 0.2262 −1.82 0.0693
##  lesions=>3  0.3818 0.2162   1.77 0.0774
##  EGFR=+ve   −0.2638 0.2544 −1.04 0.2997
##  EGFR=NA     0.4691 0.2310   2.03 0.0423
##  KPS        −0.0222 0.0078 -2.86 0.0042
##
##                Effects              Response : S
##
##  Factor              Low High Diff. Effect   S.E.    Lower 0.95 Upper 0.95
##  Age                 40  70    30    0.18716 0.29928 -0.399420  0.77373
##   Hazard Ratio       40  70    30    1.20580      NA  0.670710  2.16780
##  KPS                 90  70   −20    0.44484 0.15539  0.140280  0.74941
##   Hazard Ratio       90  70   −20    1.56020      NA  1.150600  2.11580
##  Sex - Male:Female    2   1    NA    0.62593 0.21407  0.206360  1.04550
##   Hazard Ratio        2   1    NA    1.87000      NA  1.229200  2.84480
##  ECM - No:Yes         2   1    NA    0.41089 0.22620 -0.032447  0.85424
##   Hazard Ratio        2   1    NA    1.50820      NA  0.968070  2.34960
##  lesions - >3:1-3     1   2    NA    0.38181 0.21619 -0.041907  0.80552
##   Hazard Ratio        1   2    NA    1.46490      NA  0.958960  2.23790
##  EGFR - +ve:-ve       1   2    NA   -0.26382 0.25442 -0.762470  0.23482
##   Hazard Ratio        1   2    NA    0.76811      NA  0.466510  1.26470
##  EGFR - NA:-ve        1   3    NA    0.46907 0.23096  0.016389  0.92175
##   Hazard Ratio        1   3    NA    1.59850      NA  1.016500  2.51370

##### PRPS model summary

The PRPS model (model m3) is specified below. In this model KPS is not included.

## Cox Proportional Hazards Model
##
##  cph(formula = S ~ Age + Sex + ECM + lesions + EGFR + PRPS, data = dat,
##      x = TRUE, y = TRUE, surv = TRUE, time.inc = 182.5)
##
##                       Model Tests       Discrimination
##                                            Indexes
##  Obs        140    LR chi2     35.37    R2       0.224
##  Events     111    d.f.            7    Dxy      0.354
##  Centre -0.4054    Pr(> chi2) 0.0000    g        0.671
##                    Score chi2  36.66    gr       1.956
##                    Pr(> chi2) 0.0000
##
##             Coef    S.E.    Wald Z Pr(>|Z|)
##  Age         0.0097 0.0099   0.99  0.3241
##  Sex=Female −0.5469 0.2125 −2.57  0.0101
##  ECM=Yes    −0.4287 0.2239 −1.91  0.0555
##  lesions=>3  0.3797 0.2155   1.76  0.0781
##  EGFR=+ve   −0.4445 0.2556 −1.74  0.0821
##  EGFR=NA     0.3746 0.2327   1.61  0.1075
##  PRPS       −0.0110 0.0032 −3.43  0.0006
##
##              Effects               Response : S
##
##  Factor            Low High Diff.  Effect   S.E.     Lower 0.95 Upper 0.95
##  Age               40  70    30     0.29237 0.29649  -0.288740  0.873490
##   Hazard Ratio     40  70    30     1.33960      NA   0.749210  2.395300
##  PRPS              80  40   -40     0.44086 0.12860   0.188800  0.692920
##   Hazard Ratio     80  40   -40     1.55400      NA   1.207800  1.999500
##  Sex - Male:Female  2   1    NA     0.54686 0.21248   0.130400  0.963320
##   Hazard Ratio      2   1    NA     1.72780      NA   1.139300  2.620400
##  ECM – No:Yes       2   1    NA     0.42870 0.22389  -0.010107  0.867520
##   Hazard Ratio      2   1    NA     1.53530      NA   0.989940  2.381000
##  lesions – >3:1–3   1   2    NA     0.37974 0.21552  -0.042671  0.802160
##   Hazard Ratio      1   2    NA     1.46190      NA   0.958230  2.230400
##  EGFR – +ve:-ve     1   2    NA    -0.44445 0.25565  -0.945520  0.056607
##   Hazard Ratio      1   2    NA     0.64117      NA   0.388480  1.058200
##  EGFR – NA:-ve      1   3    NA     0.37458 0.23269  -0.081493  0.830650
##   Hazard Ratio      1   3    NA     1.45440      NA   0.921740  2.294800

#### Likelihood ratio test

The likelihood ratio test is a test of model goodness to fit for two nested models. If the likelihood ratio test is not significant, it indicates that the smaller model can explain the variance in the data as well as the larger model. Hence, we can check if the addition of PRPS to a model with KPS and vice versa results in a better model fit. The likelihood ratio test of the full model versus the model with KPS is shown below

##
## Model 1: S ~ Age + Sex + ecm + lesions + EGFR + KPS + PRPS
## Model 2: S ~ Age + Sex + ECM + lesions + EGFR + KPS
##
## L.R. Chisq       d.f.          P
## 5.34288653 1.00000000 0.02080699

As we can see comparing the full model with the model including KPS, the likelihood ratio test shows that inclusion of PRPS accounts for enough variance that we can reject the null hypothesis that the coefficient for PRPS equals 0. In other words, the models are not the same.

##
## Model 1: S ~ Age + Sex + ECM + lesions + EGFR + KPS + PRPS
## Model 2: S ~ Age + Sex + ECM + lesions + EGFR + PRPS
##
## L.R. Chisq       d.f.          P
##  1.7782300  1.0000000  0.1823668

As we can see comparing the full model with the model including PRPS, the likelihood ratio test shows that inclusion of KPS does not account for enough variance that we can reject the null hypothesis that the coefficient for KPS equals 0. In other words the models are the same or that the goodness to fit is not improving with the addition of KPS to a model which already includes PRPS.

#### Model discrimination

##### C-statistic

The Harrell’s C Index is a global index for validation of a prognostic model. It is an unitless index of the rank correlation between the predicted prognosis and the actual observed prognosis. A model with a higher predictive discriminatory ability has a higher C-index. A higher value implies that the model assigns a higher probability of survival to patients with higher survival times.

***First for the full model***

##       index.orig training   test optimism index.corrected    n
## Dxy       0.3709   0.3893 0.3343   0.0549          0.3159 1000
## R2        0.2334   0.2782 0.1991   0.0791          0.1543 1000
## Slope     1.0000   1.0000 0.7898   0.2102          0.7898 1000
## D         0.0381   0.0476 0.0317   0.0158          0.0223 1000
## U        -0.0021  -0.0021 0.0047  -0.0069          0.0047 1000
## Q         0.0402   0.0497 0.0270   0.0227          0.0175 1000
## g         0.6799   0.7907 0.6182   0.1726          0.5073 1000

**Table:** C-index for the Full Model

original	corrected
0.685	0.658

***For the KPS model***

##       index.orig training   test optimism index.corrected    n
## Dxy       0.3494   0.3718 0.3210   0.0508          0.2986 1000
## R2        0.2035   0.2461 0.1732   0.0728          0.1307 1000
## Slope     1.0000   1.0000 0.7916   0.2084          0.7916 1000
## D         0.0325   0.0411 0.0270   0.0141          0.0184 1000
## U        -0.0021  -0.0021 0.0044  -0.0065          0.0044 1000
## Q         0.0346   0.0432 0.0226   0.0206          0.0140 1000
## g         0.6403   0.7454 0.5796   0.1658          0.4745 1000

**Table:** C-index for the KPS Model

original	corrected
0.675	0.649

***For the PRPS model***

##       index.orig training   test optimism index.corrected    n
## Dxy       0.3537   0.3685 0.3206   0.0478          0.3059 1000
## R2        0.2235   0.2597 0.1948   0.0649          0.1587 1000
## Slope     1.0000   1.0000 0.8260   0.1740          0.8260 1000
## D         0.0363   0.0438 0.0309   0.0128          0.0234 1000
## U        -0.0021  -0.0021 0.0029  -0.0050          0.0029 1000
## Q         0.0384   0.0459 0.0281   0.0178          0.0205 1000
## g         0.6708   0.7565 0.6194   0.1371          0.5337 1000

**Table:** C-index for the PRPS Model

original	corrected
0.677	0.653

##### Cumulative time-dependant AUC/ROC

We will use the survAUC package to calculate the time-dependant ROC curves and the AUC curves from the three model. Summary measure of the time-dependant AUC will also be calculated as iAUC. In the first setting, we will calculate the ROC using the Cumulative case/dynamic control method proposed by Uno et al.

***Full Model***

## [1] 0.7209309
##  [1] 0.6229739 0.6921212 0.7308889 0.7538652 0.7284809 0.7614379 0.7847866
##  [8] 0.7825402 0.8003692 0.8032557

***KPS Model***

## [1] 0.724338
##  [1] 0.6627907 0.7060606 0.7326667 0.7469594 0.7230628 0.7418301 0.7696351
##  [8] 0.7688476 0.7659597 0.7640083

**PRPS model**

## [1] 0.7075908
##  [1] 0.5954898 0.6753030 0.7196667 0.7438672 0.7204048 0.7558211 0.7735518
##  [8] 0.7732299 0.7976343 0.8022439

#### Reclassification scatterplots

We divided the survival outcomes by the median survival to derive two groups of patients: one with survival times of 182.5 days or less and the other whose survival times was more than 182.5 days. Patients with survival less than or equal to median were classified as having a ‘Poor Outcome’, while the others were considered to have a ‘Good Outcome’. Predicted mortality probablities at 182.5 days were then obtained from the three models. Scatter plots of predicted mortality by the full model verses the KPS model and the PRPS models were then created. The density or the number of points above and below the diagonal line was thus an indicator of the accuracy of prediction. For patients with a Good Outcome (i.e. median survival > 182.5 days), points lying below and to the right of the diagonal indicated that the full model was a better predictor of the actual survival and vice versa.

#### Risk assessment plots and IDI

Risk assessment plots are plots in which sensitivity for those with events and one specificity for those without events against the calculated risk are depicted in the same figure. The dotted lines represent the curves drawn from the data obtained from the reference model, while the solid lines represent those obtained from the new model. In our case, we designated the reference model as the KPS and PRPS models, respectively, and the new model as the full model to ascertain the added benefit of adding PRPS or KPS, respectively. The area under curve between the dotted and dashed lines are used to derive the Integrated Discrimination Improvement. In this case, the package shows the integrated discrimination improvement for both events and non-events. This makes the risk assessment plot more informative than an ROC plot because it illustrates separately how good each model is for both those with and without events. Improved performance for assigning lower risk to non-event individuals moves the reference curve (red dashed line) toward the lower-left corner (red solid line), whereas improved performance for assigning higher risk to event individuals moves the reference curve (black dashed line) towards the top-right (black-solid line).

**KPS model versus full model**

**PRPS model versus full model**

These two plots demonstrate addition of PRPS to KPS results in a slight improvement in the predictive ability with greater improvement in discriminating patients who do not have the event, that is death.

The following table shows the Integrated Discrimination Improvement statistics with 95% confidence intervals of the same.

**Table:** Integrated Discrimination Improvement

Variable	KPS versus full model	PRPS versus full model
IDI events	−0.001 (−0.0147 to 0.0127)	0.0041 (−0.0034 to 0.0117)
IDI non-events	0.0289 (0.01 to 0.0478)	0.0037 (−0.008 to 0.0154)
IDI	0.0279 (0.0049 to 0.051)	0.0079 (−0.0063 to 0.022)
Relative IDI (%)	17.61 (−2 to 37.21)	4.41 (−4.42 to 13.24)

As the table above shows the IDI is better, when PRPS is added to the KPS model. The IDI change is approximately 17%, while when KPS is added to PRPS the IDI is only 4%.

### Model calibration

Model calibration was assessed using the calibrate command of the RMS package which uses bootstrapping to get optimism corrected estimates of the estimates of predicted versus observed values.

## Using Cox survival estimates at 182.5 Days
## calibrate.cph(fit = m1, u = 182.5, B = 1000)
##
##
## n=140  B=1000  u=182.5 Day
##
##
## Mean |error|:0.0350595  0.9 Quantile of |error|:0.07233653

## Using Cox survival estimates at 182.5 Days
## calibrate.cph(fit = m2, u = 182.5, B = 1000)
##
##
## n=140  B=1000  u=182.5 Day
##
##
## Mean |error|:0.03642371  0.9 Quantile of |error|:0.06101497

## Using Cox survival estimates at 182.5 Days
## calibrate.cph(fit = m3, u = 182.5, B = 1000)
##
##
## n=140  B=1000  u=182.5 Day
##
##
## Mean |error|:0.03729151  0.9 Quantile of |error|:0.07543897

### Packages used for analysis

The following packages were used for analysis:

RStudio Team (2016). *RStudio: Integrated Development Environment for R*. RStudio, Inc., Boston, MA. http://www.rstudio.com/.

R Core Team (2017). *R: A Language and Environment for Statistical Computing*. R Foundation for Statistical Computing, Vienna, Austria. https://www.R-project.org/.

Wing MKCfJ, Weston S, Williams A, Keefer C, Engelhardt A, Cooper T, Mayer Z, Kenkel B, Team tRC, Benesty M, Lescarbeau R, Ziem A, Scrucca L, Tang Y, Candan C and Hunt. T (2017). *caret: Classification and Regression Training*. R package version 6.0-77, https://CRAN.R-project.org/package=caret.

Borchers HW (2017). *pracma: Practical Numerical Math Functions*. R package version 2.0.7, https://CRAN.R-project.org/package=pracma.

Robin X, Turck N, Hainard A, Tiberti N, Lisacek F, Sanchez J and Müller M (2011). “pROC: an open-source package for R and S+ to analyze and compare ROC curves.” *BMC Bioinformatics*, *12*, pp. 77.

Pickering “W, Endre ZH and Cairns” D (2017). *rap: Generates Risk Assessment Plot and reclassification metrics*. R package version 0.4.

Mogensen UB, Ishwaran H and Gerds TA (2012). “Evaluating Random Forests for Survival Analysis Using Prediction Error Curves.” *Journal of Statistical Software*, *50*(11), pp. 1-23. http://www.jstatsoft.org/v50/i11/.

Gerds TA (2017). *prodlim: Product-Limit Estimation for Censored Event History Analysis*. R package version 1.6.1, https://CRAN.R-project.org/package=prodlim.

Potapov S, Adler W and Schmid. M (2012). *survAUC: Estimators of prediction accuracy for time-to-event data.*. R package version 1.0-5, https://CRAN.R-project.org/package=survAUC.

Kassambara A and Kosinski M (2017). *survminer: Drawing Survival Curves using ‘ggplot2’*. R package version 0.3.1, https://CRAN.R-project.org/package=survminer.

Kassambara A (2017). *ggpubr: ‘ggplot2’ Based Publication Ready Plots*. R package version 0.1.2, https://CRAN.R-project.org/package=ggpubr.

Fox J (2016). *polycor: Polychoric and Polyserial Correlations*. R package version 0.7-9, https://CRAN.R-project.org/package=polycor.

Auguie B (2017). *gridExtra: Miscellaneous Functions for “Grid” Graphics*. R package version 2.3, https://CRAN.R-project.org/package=gridExtra.

Harrell Jr FE (2017). *rms: Regression Modeling Strategies*. R package version 5.1-0, https://CRAN.R-project.org/package=rms.

Koenker R and Ng P (2017). *SparseM: Sparse Linear Algebra*. R package version 1.76, https://CRAN.R-project.org/package=SparseM.

Harrell Jr FE, Dupont wcfC and others. m (2017). *Hmisc: Harrell Miscellaneous*. R package version 4.0-3, https://CRAN.R-project.org/package=Hmisc.

Wickham H (2009). *ggplot2: Elegant Graphics for Data Analysis*. Springer-Verlag New York. ISBN 978-0-387-98140-6, http://ggplot2.org.

Zeileis A and Croissant Y (2010). “Extended Model Formulas in R: Multiple Parts and Multiple Responses.” *Journal of Statistical Software*, *34*(1), pp. 1-13. doi: 10.18637/jss.v034.i01 http://doi.org/10.18637/jss.v034.i01.

Therneau T (2015). *A Package for Survival Analysis in S*. version 2.38, https://CRAN.R-project.org/package=survival.

Terry M. Therneau and Patricia M. Grambsch (2000). *Modeling Survival Data: Extending the Cox Model*. Springer, New York. ISBN 0-387-98784-3.

Sarkar D (2008). *Lattice: Multivariate Data Visualization with R*. Springer, New York. ISBN 978-0-387-75968-5, http://lmdvr.r-forge.r-project.org.

Daróczi G and Tsegelskyi R (2015). *pander: An R Pandoc Writer*. R package version 0.6.0, https://CRAN.R-project.org/package=pander.

Foundation TAS (2013). *SparkR: R frontend for Spark*. R package version 1.6.1.
